# Emerging Technologies in Corneal Nerve Evaluation for Dry Eye and Ocular Surface Disease: A Review

**DOI:** 10.3390/jcm15031269

**Published:** 2026-02-05

**Authors:** Chloe Shields, Natalia Davila, Alex Hattenhauer, Sakina Qazi, Anat Galor, Pragnya Rao Donthineni

**Affiliations:** 1 Bascom Palmer Eye Institute, University of Miami, Miami, FL 33136, USA; cxs1034@miami.edu (C.S.); natdavil@utmb.edu (N.D.); axh2352@med.miami.edu (A.H.); sqazi@med.miami.edu (S.Q.); 2 Department of Ophthalmology, Miami Veterans Affairs Medical Center, Miami, FL 33125, USA; 3 Shantilal Shanghvi Cornea Institute, L.V. Prasad Eye Institute, Hyderabad 500034, India

**Keywords:** corneal nerves, dry eye disease, ocular surface disease, esthesiometry, in vivo confocal microscopy, optical coherence tomography

## Abstract

Emerging evidence highlights the critical role of corneal nerves in the pathophysiology of dry eye disease (DED) and other related ocular surface disorders (OSDs). These conditions increasingly demonstrate neuropathic and neurotrophic components, wherein alterations in corneal nerve morphology and function contribute to symptomatology and disease progression. Recent advances in imaging and diagnostic modalities have enabled detailed, in vivo evaluation of corneal nerve architecture and sensory function, offering novel insights into underlying mechanisms and therapeutic responses. This review comprehensively examines current and emerging technologies for corneal nerve assessment, both structural and functional. The structural methods include in vivo confocal microscopy (IVCM), optical coherence tomography (OCT)-based nerve imaging (e.g., micro-OCT), and emerging technologies like multiphoton microscopy. The functional methods of corneal nerve assessment include advanced esthesiometers, quantitative sensory testing (QST), and functional magnetic resonance imaging (fMRI). The emerging technologies also include AI-driven analytical platforms that can be applied to both structural and functional methods. These various nerve assessment modalities can aid in delineating DED subtypes, selecting targeted treatments, monitoring nerve regeneration, and predicting treatment outcomes. By integrating structural and functional assessments, these technologies are reshaping the diagnosis, phenotyping, and management of DED and other related OSDs, paving the way for personalized therapeutic approaches.

## 1. Introduction

Dry eye disease (DED) is one of the most frequent reasons for patients seeking eye care worldwide [1,2]. According to the TFOS DEWS III report, DED prevalence estimates range from 3% to 63%, reflecting variations in study populations and diagnostic approaches [3]. These numbers not only reflect statistics but also represent the lived experiences of millions coping with daily ocular discomfort. In a community-based cohort from Melbourne, Australia (*n* = 926; 49–97 years), 17% reported ocular dryness, 26% foreign body sensation, 32% discomfort, and 50% photophobia [4], suggesting that chronic, bothersome ocular surface symptoms are globally prevalent, diminishing visual comfort and quality of life for many. Numerous studies have suggested the role of corneal sensitivity in symptoms of DED. A Canadian study involving 18 patients with Sjögren’s disease (SjD), showed an inverse relationship between corneal sensation assessed using the Cochet–Bonnet esthesiometer and ocular surface staining (OSS) (fluorescein: r = −0.35; lissamine green: r = −0.37, *p* = 0.03 for both), indicating lower sensations in individuals with more ocular surface disruption. Alternatively, a Finnish study of 20 SjD patients noted lower Belmonte aesthesiometer-assessed corneal detection thresholds compared to 10 controls (54.5 ± 40.1 mL/min vs. 85.0 ± 24.6 mL/min, *p* = 0.03), indicating corneal hypersensitivity in some individuals. These thresholds negatively correlated with symptom severity (ocular surface disease index (OSDI): r = −0.46, *p* < 0.01), indicating a higher symptom burden in individuals with corneal hypersensitivity [5]. These data highlight that both decreased and increased corneal sensations can contribute to disease manifestations in DED and other ocular surface disorders (OSDs), suggesting that corneal nerves play a crucial—yet heterogeneously altered—role even within the same disease.

At the center of ocular surface homeostasis lies the cornea’s extraordinary sensory innervation, with approximately 7000 terminals/mm^2^, making it 300 to 600 times more sensitive than skin. These corneal nerves are essential for blink reflexes, trophic support of the epithelium, modulation of inflammation, and maintenance of tear secretion [6,7]. Damage or dysfunction of these nerves can contribute to both the onset and perpetuation of DED and related OSDs. Corneal nerve damage or dysfunction can result in either reduced sensations causing neurotrophic keratopathy (NK) [7] or by producing neuropathic corneal pain (NCP) associated with hyperalgesia and allodynia [8,9]. Consequently, DED and other OSDs are increasingly being viewed as neuroinflammatory syndromes with substantial neuropathic as well as neurotrophic involvement [10]. While triggers such as surgical trauma, sustained tear hyperosmolarity, or chronic surface inflammation can initiate or aggravate the corneal nerves, sensitization of peripheral nociceptors and central pain pathways, sometimes accompanied by abnormal nerve regeneration (e.g., microneuroma formation), may also occur without clear external triggers [9,11,12,13,14]. These neural adaptations help explain why many patients experience severe dryness, burning, or ocular pain despite only modest clinical signs on examination [11,15].

**Figure 1:**
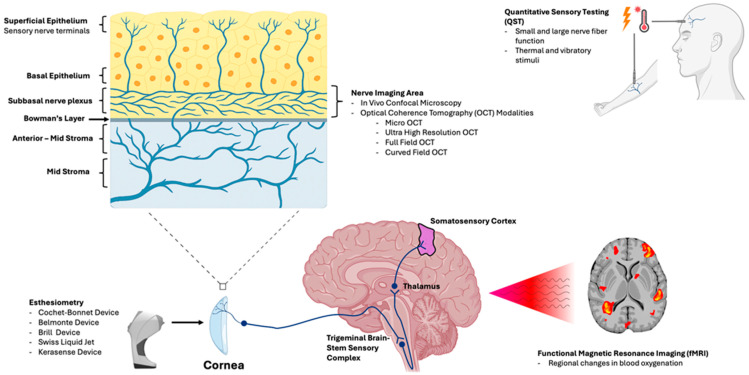
Illustration linking each modality to the anatomic level evaluated. In vivo confocal microscopy (IVCM) and optical coherence tomography (OCT)-based approaches assess corneal nerve structure and morphology, particularly within the subbasal nerve plexus (SNP). Corneal esthesiometry and quantitative sensory testing (QST) quantify sensory thresholds and peripheral somatosensory function. Central sensory processing is evaluated along the trigeminal pathway (brainstem to thalamus and somatosensory cortex), using functional magnetic resonance imaging (fMRI), to characterize pain-related neural activation.

As neurosensory dysfunction both drives symptoms and limits treatment response, assessment of peripheral and central nerves has become increasingly relevant in recent times. With advancements in sensory testing and imaging, clinicians now have access to integrated tools that offer objective insight into both the structure and function of corneal nerves. Structural evaluation can be achieved using in vivo confocal microscopy (IVCM), which is used to visualize the subbasal nerve plexus (SNP) at subcellular resolution [16,17], and using optical coherence tomography (OCT), which allows for non-contact imaging of nerve morphology [18,19]. Alternately, functional assessment includes advanced contact and non-contact esthesiometers that measure stimulus-specific thresholds [20,21], quantitative sensory testing (QST) that assesses both peripheral and central sensitization [22], and functional magnetic resonance imaging (fMRI) that captures brain-level processing of ocular pain [23]. Together, these modalities can evaluate both structure and function of the corneal nerves and associated pathways, enabling more precise phenotyping of DED and related OSDs, monitoring of nerve regeneration, and prediction of therapeutic outcomes. Figure 1 gives us an overview of how each modality aligns with the anatomic level evaluated.

Thus, this narrative review synthesizes established and emerging technologies for the structural and functional evaluation of corneal nerves and highlights the clinical utility of these tools in diagnosing and subtyping DED, monitoring nerve regeneration, and guiding personalized treatment strategies. In this review, we classify corneal nerve assessment technologies based on the level of clinical adoption and validation. “Established” modalities are those with widespread clinical availability, routine clinical use, and substantial data supporting diagnostic or monitoring utility. “Emerging” technologies include (1) novel devices not yet widely deployed clinically, (2) established platforms being applied in new ways (e.g., OCT adapted for SNP visualization), and (3) analytical advances (e.g., AI advances) that enhance interpretability or scalability but remain limited by external validation, regulatory status, and implementation barriers. This classification is intended as a conceptual framework rather than an exhaustive label for every modality discussed.

## 2. Corneal Nerve Biology and Pathophysiologic Relevance

The cornea is the most densely innervated tissue in the human body with a nerve density that far exceeds that of skin, resulting in exquisite sensitivity and rapid reflexes that preserve ocular surface homeostasis [24]. The corneal neural network originates from the ophthalmic branch of the trigeminal nerve, with afferent neuronal cell bodies located in the trigeminal ganglion and central projections extending to the trigeminal brainstem sensory complex that ultimately relay pain signals to cortical and subcortical brain centers [24,25]. Nerve processes enter the peripheral cornea as thick stromal nerve bundles, typically 50–70 bundles arranged in a radial pattern from the limbus, which branch extensively as they course anteriorly through the stroma, progressively reducing in diameter. Upon reaching the anterior third of the stroma, nerve fibers penetrate Bowman’s layer and form the SNP [24,25]. The SNP is arranged in a vortex-like configuration around the corneal apex, transitioning to a more radial pattern in the peripheral cornea [25]. From the SNP, fine nerve terminals ascend through the epithelium as free nerve endings [25]. These nerve terminals house various types of sensory receptors, known as nociceptors, that are predominantly of 3 types: (i) mechanoreceptors (~20%), (ii) polymodal (~70%), and (iii) cold thermoreceptors (~10%) [26]. Mechanoreceptors respond to mechanical stimuli alone [27]. In contrast, polymodal receptors respond to mechanical, acidic/basic, and heat stimuli, and cold receptors sense temperature change [27,28]. The terminal membranes of the polymodal nociceptors house transient receptor protein vanilloid 1 (TRPV1) antagonist channels that detect noxious stimuli (chemicals, pH, heat), and the terminal membranes of the cold thermoreceptors house TRPM8 that detects temperature drop/evaporation [25,29,30].

The structure and function of corneal nerves play a major role in DED and related OSDs, encompassing both neurotrophic and neuropathic mechanisms that, while distinct, can often overlap [31]. A neurotrophic state, driven by reduced nerve density, impaired neurotrophic support, and defective reflex tearing, disrupts epithelial maintenance and healing [32], and in advanced stages manifests as NK with epithelial breakdown, stromal changes, and ulceration despite minimal pain [33]. Conversely, aberrant nerve regeneration and dysregulated sensory signaling can lead to NCP [34], in which peripheral and/or central sensitization produce spontaneous pain or pain disproportionate to ocular surface findings. Peripheral sensitization occurs following peripheral nerve injury or local inflammation and leads to a reduced activation threshold of nociceptive neurons in the periphery, resulting in heightened neuronal responsiveness [12]. In contrast, central sensitization involves neuroplastic changes within the central nervous system (CNS) that develop from persistent peripheral input and repeated activation of spinal and supraspinal nociceptive pathways, including the somatosensory cortex, thalamus, and amygdala [11,13]. These central changes promote neuronal hyperexcitability, which manifests clinically as hyperalgesia, allodynia, and the transition to a chronic pain state [9,12,35]. In parallel, autonomic nerve dysfunction may contribute to ocular pain amplification through abnormal sympathetic–parasympathetic signaling, neuroimmune modulation, and disordered neurovascular responses and may help explain systemic comorbidities and widespread sensory abnormalities observed in a subset of patients with NOP [36,37]. In this review, we use the terminology neuropathic/nociplastic ocular pain (NOP) when referring to pain generated by peripheral, central, or autonomic nerve abnormalities and consider NCP a subtype of NOP that refers to pain driven by peripheral corneal nerve abnormalities.

## 3. Structural Assessment Technologies for Corneal Nerves

Structural imaging technologies provide essential insight into the morphology and integrity of corneal nerves and enable direct visualization of the subbasal nerve plexus, quantification of nerve density and branching, and assessment of epithelial and stromal architecture. Clinically, structural imaging can aid in assessing nerve morphology, monitoring disease progression, and evaluating therapeutic efficacy. Among these, IVCM and OCT-based nerve imaging are the most widely used, while emerging tools such as multiphoton microscopy, optical coherence microscopy, and ocular surface analyzers are expanding the range of measurable parameters in both research and clinical practice. Table 1 summarizes all the structural assessment technologies covered in this review.

### 3.1. IVCM

IVCM is a noninvasive imaging modality that allows real-time, high-resolution imaging of the human cornea, enabling visualization of all the layers. IVCM is currently the most established modality for in vivo corneal nerve visualization but remains limited by equipment availability and specialized expertise. The technique relies on the principle of confocality, where a focusing objective and a pinhole aperture exclude out-of-focus light, producing a thin optical section with a lateral resolution of ~1–2 µm and axial (depth) resolution of ~10–26 µm depending on the device [17]. Through its ability to deliver histologic-level detail in vivo, IVCM has emerged as the definitive tool for quantitative and longitudinal assessment of corneal nerves. Three principal types of IVCMs are commercially available: the Tandem Scanning Confocal Microscope (TSCM; Tandem Scanning Corp., Reston, VA, USA), the Nidek Confoscan-4 (Nidek Technologies Srl, Padova, Italy), and the Heidelberg Retinal Tomograph with Rostock Corneal Module (HRT-RCM, Heidelberg Engineering, GmBH, Dossenheim, Germany) [38].

The earliest device, the TSCM, introduced by Petráň et al. in 1968 [47], used a spinning Nipkow disk with pinholes in Archimedean spirals for high axial/lateral resolution and gave the first high-contrast optical sections of living corneas; however, <1% of light reached the tissue due to scatter, requiring powerful sources and sensitive cameras [40]. The slit-scanning confocal microscope (SSCM), conceived by Svischev in the late 1960s and applied to human corneas by Masters and Thaer in 1994 [41], employed vertical slit apertures for brighter images and better epithelial/stromal visualization than TSCM [40]. The widely used Confoscan-4 enables fast, non-contact cell/nerve imaging but with lower resolution and less distinct subbasal nerves than laser systems [39]. Conversely, laser scanning confocal microscope (LSCM) uses a 670 nm diode laser with galvanometric mirrors [39]. The HRT-RCM (Heidelberg Engineering), the leading corneal LSCM, combines a 0.9 NA 63× objective for ~1–2 µm lateral/~4 µm axial resolution, near-histologic subbasal nerve imaging, and sequence-scan mode for dynamic depth-specific imaging, enabling in vivo visualization of the corneal SNP with near-histologic clarity. With these capabilities, the HRT-RCM can capture detailed morphological features like nerve beading, microneuromas, and branching patterns, as well as immune and inflammatory cells within the subbasal layer. Although this technique needs contact and specialized operator training, its high contrast and reproducibility make it one of the preferred methods for structural assessment of corneal nerves in DED, NCP, and NK [39,40].

**Figure 2:**
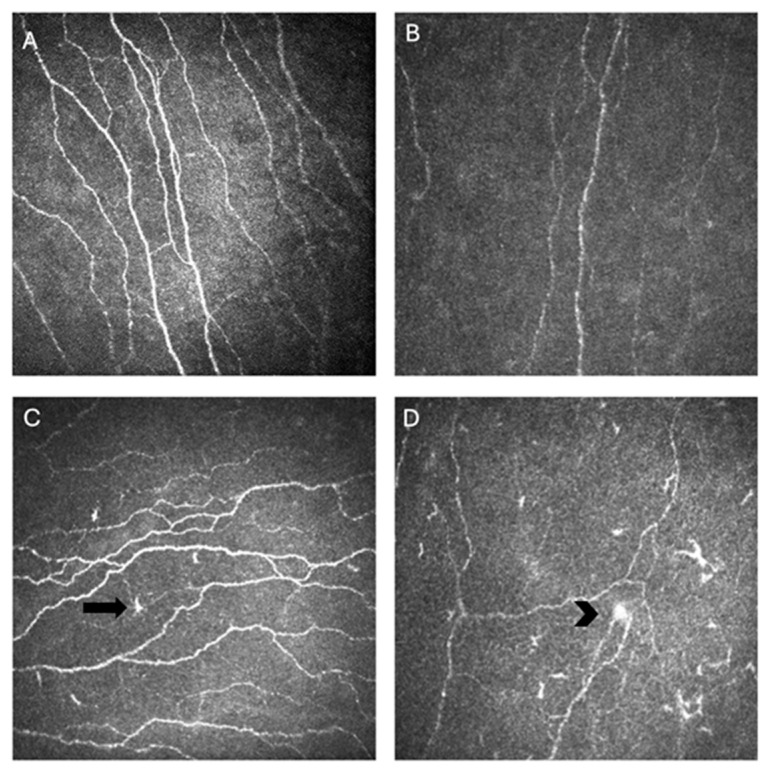
In vivo confocal microscopy (IVCM) images of corneal nerves showing normal nerve parameters (**A**), decreased nerve density in an individual with Sjögren’s disease (SjD) (**B**), activated dendritic cells (arrow) and tortuosity in an individual with SjD (**C**), and a microneuroma (arrowhead) with activated dendritic cells and decreased nerve density (**D**).

The most widely reported metrics to quantify corneal nerve morphology using IVCM include corneal nerve fiber length (CNFL), expressed as total length of nerve fibers per square millimeter; corneal nerve fiber density (CNFD), defined as number of nerve fibers per square millimeter; and corneal nerve branch density (CNBD), capturing the frequency of bifurcations per square millimeter. Additional parameters such as nerve tortuosity, beading frequency, and microneuroma count can capture finer variations in nerve architecture and regenerative patterns [40]. Image analysis software, like Automated Cell Count (ACC) Metrics (Heidelberg Engineering, GmBH), allows automatic quantification of these metrics from single or multiple images [17]. Together, these metrics provide a standardized framework for assessing the structural integrity and remodeling of the corneal SNP across disease states and therapeutic interventions (Figure 2) [40].

CNFD is one of the most frequently assessed parameters in IVCM and has shown strong associations with DED symptom severity. An Indian study stratified individuals with evaporative dry eye (EDE) by symptom intensity into normal to mild (OSDI < 23; *n* = 29) and moderate to severe (OSDI > 23; *n* = 23) groups, along with healthy controls (*n* = 43). CNFD was significantly reduced in those with moderate to severe symptoms compared to controls (26.2 ± 1.0 nerves/mm^2^ vs. 28.6 ± 0.8 nerves/mm^2^; *p* = 0.04), whereas no difference was seen in the mild group (27.9 ± 0.7 nerves/mm^2^; *p* > 0.05) [48]. Consistent trends were reported when comparing DED subtypes. In a French cohort of patients with NCP, defined as ocular pain with minimal surface staining (Oxford score ≤ 1), CNFD was significantly lower in those with meibomian gland dysfunction-related NCP (MGD-NCP) compared to controls, while autoimmune-associated NCP (AIDE-NCP) showed no significant difference [49]. Collectively, these findings demonstrate that CNFD can reflect both the severity and etiology of DED, suggesting potential use in phenotyping patients with overlapping nociceptive and neuropathic features.

Beyond CNFD, other quantitative metrics such as CNFL and CNBD further enhance the structural characterization of the SNP. An Italian study comparing DED patients (*n* = 39) to healthy controls (*n* = 30) found that both CNFL and CNBD were reduced in the DED group (12.6 ± 4.4 vs. 14.5 ± 2.9 mm/mm^2^, *p* = 0.024; 25.6 ± 20.1 vs. 37.6 ± 21.5 n/mm^2^, *p* = 0.01) [50]. Furthermore, a US phase IV clinical trial of treatment for DED associated with MGD not only found that DED patients (*n* = 54) had lower CNFL compared to healthy controls (*n* = 27; 17.06 ± 5.78 mm/mm^2^ vs. 23.68 ± 3.42 mm/mm^2^, *p* = 0.001), but also that those with low baseline CNFL (<16.84 mm/mm^2^) had no significant improvement in signs or symptoms following treatment with either artificial tears or loteprednol. However, those with normal baseline CNFL values (≥16.84 mm/mm^2^) had significant changes in signs and symptoms following treatment (OSDI severity 58.3 ± 23.2 vs. 40.3 ± 14.4, *p* = 0.04; corneal fluorescein staining (CFS) 5.5 ± 1.8 vs. 3.4 ± 2.4, *p* = 0.01) [51]. Together, these studies emphasize that CNFL and CNBD serve not only as diagnostic indicators but also as prognostic markers that can predict treatment response in DED.

Across various DED phenotypes, IVCM consistently reveals characteristic patterns of subbasal nerve alteration, with increased tortuosity emerging as one of the most reproducible findings. In a US study comparing SjD patients (*n* = 22), non-Sjögren’s dry eye (NSDE; *n* = 12), and healthy controls (*n* = 5), tortuosity graded using the Oliveira-Soto and Efron (OSE) scale was higher in both the SjD and NSDE groups (2.69 ± 0.69 and 2.78 ± 0.75, respectively) compared to controls (2.13 ± 0.48; *p* < 0.05 for both) [52]. In a French NCP cohort, tortuosity graded from 0 to 4 was significantly higher in the AIDE-NCP group (*n* = 7) compared to controls (*n* = 10; *p* < 0.05), whereas the MGD-NCP group (*n* = 11) showed no difference relative to either group [49]. Another French study comparing SjD (*n* = 71), MGD (*n* = 20), and healthy controls (*n* = 20) found that OSE-graded tortuosity was higher in patients with SjD compared to the healthy control (2.3 ± 0.6 vs. 1.8 ± 0.3; *p* < 0.001) and MGD (2.0 ± 0.4; *p* = 0.025) groups [53]. Collectively, these data reinforce tortuosity grading as a reliable morphologic marker that can distinguish DED subtypes. It may be most relevant in patients with SjD, possibly reflecting underlying differences in inflammatory and nerve-related mechanisms. Regarding less of the clinical context, tortuosity may be best classified as a supportive biomarker, as there are few data to suggest that it can serve independently as a diagnostic tool. However, in combination with other nerve markers, it may hold greater diagnostic value, with one Chinese cross-sectional study reporting an AUC of 0.91 (95% CI 0.86–0.95) for a set of four quantified nerve parameters including tortuosity. They also found that tortuosity (based on the multiple segmentation algorithm) was positively associated with OSDI scores (β = 17.7, *p* < 0.001) and increased with increasing clinical severity of DED, assessed using a modified DEWS grading standard (3.34 ± 0.03 in grade 2 DED vs. 3.09 ± 0.04 in grade 1 DED, *p* < 0.05), suggesting that it may be an indicator of disease severity [54]. Elsewhere, data are contradictory, with some studies showing no association with OSDI scores or objective DED signs such as TBUT or Schirmer’s test [52,53]. Therefore, while corneal nerve tortuosity has proven to be a supportive phenotypic feature of DED, particularly in SjD populations, its diagnostic and prognostic utility remains uncertain.

Beyond nerve length and branching, IVCM can also visualize focal structural abnormalities such as microneuromas, which are bulb like enlargements of subbasal nerve endings that indicate injury and abnormal regeneration. Several studies have reported higher frequencies of microneuromas among specific DED subtypes. An Indian cross-sectional study of individuals with DED symptoms (*n* = 93) compared to healthy controls (*n* = 27) found that microneuromas were present in 11.1% of controls and 85.7% of those with OSDI scores that outweighed signs like TBUT and Schirmer [55]. Similarly, a French study of NCP patients reported that the mean number of microneuromas per patient was significantly higher in both MGD-NCP and AIDE-NCP groups compared to healthy controls [49]. Conversely, a US study evaluating microneuromas in individuals with DED symptoms and prior refractive surgery (*n* = 16), DED symptoms without refractive surgery (*n* = 119), and controls (*n* = 18) found similar frequencies across the groups (6.3, 21.8, and 11.1% respectively, *p* = 0.22) [56]. Together, these findings suggest that microneuromas may be more frequent in certain DED subtypes, particularly when NCP features are present, but further studies using standardized imaging and quantification methods are needed before establishing them as reliable biomarkers of disease. They may also be considered supportive markers, though weaker and less reliable than other nerve parameters, and it remains unclear which patient population their detection is most applicable to.

Thus, IVCM allows clinicians to characterize DED beyond conventional tear film parameters. Distinctive morphologic patterns such as reduced CNFD, altered branching, microneuroma formation, or increased dendritic cell infiltration can help differentiate nociceptive DED from neuropathic or mixed pain phenotypes where symptom severity is disproportionate to clinical signs, supporting a diagnosis of NCP rather than tear film-mediated discomfort. Quantitative nerve metrics can also serve as objective markers to guide and monitor treatment, identifying patients more likely to benefit from neuroregenerative or neuromodulatory therapies such as autologous serum tears or oral neuromodulators, while avoiding unnecessary escalation of topical anti-inflammatory regimens.

### 3.2. Optical Coherence Tomography (OCT)

The OCT was first introduced in 1991 by Dr. James Fujimoto and colleagues at the Massachusetts Institute of Technology, demonstrating the technology’s ability to generate cross sectional optical images of biological tissues, including the retina [57]. It operates by directing low coherence light toward tissue and measuring the intensity of backscattered signals, similar to ultrasound detecting reflected sound waves to visualize internal structures [57]. Each axial reflectivity profile, termed an A scan, represents a single depth measurement, and multiple adjacent A scans are compiled laterally to create a cross-sectional B scan. The earliest OCT systems achieved axial resolutions of 10 to 20 μ, but subsequent innovations like spectral-domain and swept source detection dramatically improved acquisition speed, penetration depth, and image fidelity, expanding applications to front of the eye [57]. The first demonstration of anterior segment OCT (AS-OCT) was published by Izatt et al. in 1994, showing high-resolution corneal cross-sectional images with clear delineation of epithelium, stroma, and endothelium, with reliable pachymetry measurements [58]. Early broadband systems, however, lacked the resolving power to visualize cellular scale structures such as corneal nerves. Although OCT is widely established in ophthalmic diagnostics, the emergence of high-resolution and ultra-high-resolution (UHR) OCT systems in the late 2010s allowed in vivo imaging of the SNP.

One of the first OCT types capable of resolving fine corneal microstructures was the UHR OCT. Werkmeister et al. in 2017 employed a spectral domain UHR OCT system driven by a broadband Ti:sapphire laser centered near 800 nm, achieving ~1.2 μm axial and 20 μm lateral resolution at 140 kilohertz [42]. Within the anterior stroma and subbasal region, the system achieved sufficient contrast to visualize linear, hyper-reflective structures consistent with corneal nerve bundles, establishing that UHR OCT can noninvasively detect fine neural features [42]. Advancements in geometric OCT design led to the development of full-field (FF) OCT, which replaces sequential point scanning with parallel, camera-based en face acquisition. Using this approach, a French study presented the first in vivo human visualization of the SNP and stromal nerves, achieving approximately 1.6 μm lateral and 7.7 μm axial resolution across a 1.26 × 1.26 mm field, producing confocal like en face maps of corneal innervation within seconds [18]. These images were later refined using curved field (CF) OCT, which optically matches the focal plane to the cornea’s curvature so that each en face slice remains confined to a single anatomical layer. Another French study demonstrated this enhancement, generating curvature corrected fields of 1.13 × 1.13 mm with cell level detail and nerve calibers of 2 to 4 μm, captured at acquisition speeds exceeding 0.6 billion pixels per second [19]. This configuration yielded broader, sharper, and layer pure visualizations of corneal innervation suitable for quantitative mapping of the SNP.

The highest spatial resolution to date has been achieved with micro-OCT, also termed optical coherence microscopy (OCM), though its use is confined to animal and ex vivo corneal models currently. A US- and Germany-based collaborative study employed a broadband source and high numerical aperture optics to achieve approximately 1 μm axial × 1.5 μm lateral resolution, enabling visualization of individual epithelial cells, the SNP, and stromal nerve bundles within 1 × 1 mm volumes [43]. The resulting three-dimensional reconstructions captured fine nerve branching and epithelial to nerve interfaces not discernible with other OCT modalities. While micro-OCT remains preclinical, its unprecedented resolving power positions it as a potential future platform for quantitative, non-contact assessment of corneal innervation in DED.

Building on these advances, polarization-sensitive (PS) OCT introduces an additional source of contrast by detecting tissue birefringence, which reflects the orientation and organization of fibrous microstructures such as nerves. A US study demonstrated polarization-dependent OCM capable of capturing in vivo en face images of human SNP, where polarization contrast accentuated nerve visibility without altering the underlying spatial resolution [44]. Similarly, the US- and Germany-based collaborative study, as previously mentioned, also applied PS micro-OCT to animal and ex vivo corneas, producing birefringence maps that highlighted nerve trajectories within the surrounding stromal matrix [43]. Together, these studies illustrate how polarization contrast can enhance nerve delineation and orientation mapping, complementing structural detail achieved with intensity-based imaging.

### 3.3. Other Structural Technologies

Other imaging methods have been used to visualize corneal nerves, though most have been validated primarily in animal models. Multiphoton laser microscopy, which excites fluorescent molecules using two low energy photons, enables high-resolution three-dimensional imaging of living tissue while minimizing collateral damage. In living mice stained with vital dyes, a Chinese study demonstrated clear in vivo visualization of the corneal layers and subbasal nerve plexus, confirming the technique’s capacity to resolve fine neural structures within the intact cornea [45]. Extending this approach to disease modeling, a US study used intravital multiphoton microscopy in a murine DED model to dynamically quantify immune–nerve interactions. While not looking at the nerves themselves, they found significantly increased density of corneal dendritic cells in the limbus and peripheral cornea of mice with DED as evidenced by CFS and tear volume, compared to controls without DED (limbus 437.5 ± 45.1 vs. 260.4 ± 30.9, *p* = 0.01; peripheral 243.8 ± 32.9 vs. 127.5 ± 23.2, *p* = 0.021) [46]. These results demonstrate that multiphoton microscopy can capture real-time structural changes on the ocular surface, supporting its utility as a preclinical research platform for studying corneal nerves and associated immune activity during ocular surface inflammation. However, its translation into routine clinical use remains limited by practical, technical, and validation barriers similar to those faced by OCT imaging.

## 4. Functional Assessment Technologies

Sensory function testing is particularly relevant in DED and OSD because altered detection thresholds correlate with distinct symptom clusters, epithelial disease severity, and patterns of nerve alterations documented by IVCM and clinical scoring systems [59,60,61,62]. Psychophysical detection and pain thresholds to mechanical, thermal, and chemical stimuli quantify somatosensory function revealing subclinical nerve dysfunction before overt clinical signs emerge [21,63]. Practical clinical applications include diagnosis, phenotyping patients into neuropathic vs. neurotrophic categories, monitoring disease progression, objectively measuring treatment response in therapeutic trials, and stratifying patients for targeted interventions [59,60,61,62]. Table 2 summarizes all the functional assessment technologies covered in this review.

### 4.1. Esthesiometry

Esthesiometry delivers controlled stimuli to quantify corneal sensation and thereby assess the functional integrity of corneal innervation, complementing structural imaging modalities [20,59]. Esthesiometry captures functional deficits that may not be apparent on structural imaging alone, providing further information on nerve health [59,60]. Several types of esthesiometers are available, differing in the nature and control of the applied stimulus. Conventional esthesiometers, like the Cochet–Bonnet and Belmonte devices, use calibrated mechanical and pneumatic stimuli, respectively, to measure corneal sensitivity thresholds with high precision. Recently developed non-contact esthesiometers based on gas jets, air puffs, or microfluidic systems enhance reproducibility and minimize epithelial disruption. The Cochet–Bonnet esthesiometer represents the classical contact mechanical esthesiometer, considered the clinical standard that uses a variable length nylon monofilament to deliver a calibrated bending force to the corneal surface [20,62]. The filament length is progressively reduced until the patient no longer perceives the stimulus, yielding a threshold expressed as the longest filament length that evokes sensation or converted to force units; lower threshold lengths indicate reduced corneal sensitivity [20,62]. The Cochet–Bonnet esthesiometer remains widely used in NK monitoring and clinical research due to its simplicity, portability, low cost, and extensive historical datasets that enable comparison across studies [20,62].

Gas esthesiometers represent a major technological advancement, generating controlled air jets whose flow rate, temperature, and chemical composition (CO_2_ admixture) can be independently adjusted to enable quantitative mechanical, thermal, and chemical stimulation of the corneal surface [21,64,75]. The Belmonte esthesiometer employs a computerized system that delivers air pulses through a fine nozzle positioned at a fixed distance from the cornea, with stimulus intensity controlled by adjusting flow rate, temperature, and CO_2_ concentration [21]. This multimodal capability enables selective activation and threshold determination of distinct corneal nociceptor subtypes: pure mechanical stimulation activates mechanoreceptors, cold thermal stimulation selectively activates cold thermoreceptors (TRPM8-expressing C-fibers), heat stimulation activates heat-sensitive polymodal nociceptors, and chemical stimulation activates polymodal nociceptors via acid-sensing ion channels and TRPV1 [21,64,75]. Quantitative parameters measured include detection thresholds for each stimulus modality, pain thresholds, and stimulus-response curves that characterize nociceptor sensitivity profiles [21,64]. Neurotrophic patients typically show elevated thresholds across all modalities reflecting loss of functional innervation, whereas NCP patients may exhibit reduced pain thresholds or abnormal thermal/chemical responses indicating hypersensitivity [63,64,76].

Both Cochet–Bonnet and Belmonte esthesiometers have been widely studied in DED to characterize somatosensory variability and phenotype-specific dysfunction [77]. In a study involving 403 South Florida veterans with DED symptoms (Dry Eye Questionnaire 5 (DEQ-5) ≥ 6), the mean corneal detection threshold measured using the Belmonte esthesiometer was 87 ± 46 mL/min (10–90% range = 40–145 mL/min). Of them, 24% of individuals tested outside this range, 13% had hypersensitivity, and 11% had hyposensitivity, and hypersensitivity was associated with greater ocular pain intensity [78]. Similarly, a US study of 129 DED patients, lower corneal mechanical detection measured using a Belmonte esthesiometer correlated with worse dry eye symptoms and higher ocular pain scores [64]. These results highlight heterogeneity in corneal sensitivity and its relation to DED symptoms. Esthesiometry has been also been applied across diverse OSDs including NK, post-surgical trigeminal injury, contact lens-related changes, glaucoma medication toxicity, and ocular graft-versus-host disease (GVHD) to guide clinical decision making and monitor therapeutic interventions [59,60,61,62]. For instance, epidemiologic series of NK and ocular GVHD have used Cochet–Bonnet to quantify hypoesthesia and relate corneal sensitivity to epithelial disease severity and clinical outcomes [63,79].

Newer and advanced esthesiometers have emerged to address limitations of traditional devices and expand functional assessment capabilities. The Brill esthesiometer (Brill Engines, Spain) is a non-contact device that delivers controlled air pulses with adjustable flow rates, offering improved standardization, patient comfort, and reduced infection risk compared to contact methods. Validation studies report good repeatability and moderate to strong correlation with Cochet–Bonnet measurements, though raw threshold values are not directly interchangeable between devices [65,66]. The Swiss Liquid Jet esthesiometer is a noninvasive device that projects tiny droplets of isotonic saline onto the central cornea to quantify corneal sensitivity. It primarily delivers a mechanical stimulus by varying droplet pressure over 100–1500 mbar with 1 mbar precision, and has broader dynamic range than traditional devices with the capability for mechanical, thermal, and chemical-dominant stimulation paradigms, delivering standardized non-contact stimulus improving inter-operator concordance and detecting subtle threshold changes [67,68]. Kerasense (Arcion Therapeutics, Baltimore, MD, USA) represents a novel single-use, mechanical esthesiometer that measures corneal sensitivity in millimeters and has demonstrated good test–retest repeatability to identify subclinical NK in at-risk populations (diabetes, glaucoma, post-refractive surgery) with high sensitivity and specificity [69].

Other emerging approaches include automated multi-jet air systems with software-controlled micro-blowers and multiple nozzles that enable spatially and temporally separated stimuli, measured airflow forces in the micro-Newton range, and sophisticated QST including spatial summation and temporal integration studies [80,81].

### 4.2. Cutaneous QST

Cutaneous QST is a psychophysical method used to evaluate the functional integrity of somatosensory pathways by delivering controlled mechanical, thermal, or vibratory stimuli to the skin and recording the subject’s sensory or pain responses. The technique assesses both small- and large-fiber function and can identify alterations in sensory processing that suggest peripheral or central sensitization [70]. It has been applied to cutaneous sites such as the forehead and forearm in patients with DED and NOP to assess systemic somatosensory abnormalities [71,82]. By quantifying sensory thresholds (e.g., detection and pain thresholds), temporal summation (the increase in perceived pain with repeated stimulation), and aftersensations (pain persisting beyond stimulus removal), QST provides objective, reproducible measures of pain sensitivity and modulation. It complements ocular assessments and distinguishes localized corneal pathology from generalized somatosensory dysregulation, improving phenotyping of patients across neurotrophic–neuropathic pain spectrum [22]. Reduced pain thresholds/elevated responses confined to the stimulated region are more consistent with peripheral nociceptor sensitization, whereas temporal summation and aftersensations reflect CNS changes (central sensitization) with augmented pain processing and reduced descending inhibition [72,83].

Numerous studies have shown correlation between QST measures and DED and NOP symptoms. A US study of 118 veterans with a variety of DED symptoms and signs found that hot pain temporal summation (HPTS) values at the forearm positively correlated with neuropathic-like pain qualities, including ocular burning (r = 0.25, *p* = 0.007), wind sensitivity (r = 0.22, *p* = 0.02), and overall Neuropathic Pain Symptom Inventory (NPSI)-Eye score (r = 0.26, *p* = 0.004). In addition, HPTS aftersensation ratings correlated with burning (r = 0.20, *p* = 0.03), wind (r = 0.18, *p* = 0.06) and light sensitivity (r = 0.22, *p* = 0.02), and NPSI-Eye total score (r= 0.28, *p* = 0.002) [22]. A larger cohort of 326 veterans extended these findings, showing that aftersensation intensity to thermal (hot and cold) stimuli at the forehead and forearm positively associated with symptom–sign discordance, suggesting that patients with severe symptoms but minimal ocular surface findings exhibit abnormal persistence of thermal pain [82]. Furthermore, a study of 224 individuals with DED symptoms (DEQ5 ≥ 6) found that those with persistent ocular pain despite topical anesthesia exhibited greater sensitivity to evoked pain at both the forehead (hot pain threshold (HPT): 9.7 (4.2) vs. 12.5 (4.2), *p* = 0.006; pain intensity at HPT: 58.3 (26.6) vs. 45.2 (25.6), *p* = 0.03) and forearm (cold pain threshold (CPT): 13.1 (9.6) vs. 19.6 (9.9), *p* = 0.006; pain intensity at CPT: 49.4 (23.5) vs. 35.1 (26.1), *p* = 0.02), supporting the role of central amplification in sustaining ocular pain even when corneal input is transiently silenced [71]. Finally, in 235 individuals with DED (DEQ5 ≥ 6), pain due to light (0–10 scale) predicted the presence of thermal pain aftersensations at the forearm, highlighting a clinically accessible marker for identifying patients with a central NOP phenotype [84].

Building on thermal QST findings, several studies have applied mechanical stimuli to assess somatosensory function. A study of 43 LASIK recipients found that lower mechanical pain thresholds (assessed using pin prick stimulators) at the forehead correlated with higher baseline ocular pain (r = −0.40, *p* = 0.01) and those with presence of mechanical aftersensations pre-operatively at the forearm had higher DED symptoms at six months (DEQ5 scores: 8.0 ± 1.9 vs. 5.0 ± 5, *p* < 0.005). These data suggest that cutaneous mechanical hypersensitivity and pain persistence can serve as risk markers for persistent post-surgical ocular pain [37].

Across studies, thermal temporal summation and aftersensations consistently correlate with NOP and symptom–sign discordance, reinforcing the concept that, for a subset of patients, DED symptoms may reflect generalized somatosensory dysregulation rather than isolated ocular surface pathology [22,37,71,82]. This central sensitization framework provides a neurobiological explanation for persistent pain in the absence of clinical signs and highlights the value of cutaneous QST as a noninvasive tool for identifying patients with a central pain phenotype. Thus, QST-derived sensory profiles can help clinicians interpret symptom–sign mismatch, anticipate postoperative pain risk, and guide management strategies that include central pain-modulating therapies (e.g., neuromodulators, cognitive-behavioral interventions, or neurostimulation) in addition to conventional ocular surface treatments.

### 4.3. Functional Magnetic Resonance Imaging (fMRI)

fMRI provides an objective, noninvasive window into the neural correlates of ocular pain by detecting regional changes in blood oxygenation that reflect neuronal activity in the brain. It allows characterization of altered brain responses to sensory stimuli and identification of central pain mechanisms underlying chronic NOP [37,73,74]. In individuals with DED and NOP, fMRI has revealed distinct activation patterns in pain-related cortical and subcortical regions. A study of 16 South Florida veterans (8 with NOP and photophobia ≥ 6 months and 8 controls) had participants alternately viewing bright (white) and dark (black) screens during fMRI scanning. The NOP group demonstrated greater blood oxygen level-dependent (BOLD) activation in the primary somatosensory cortex (S1), insular cortex, and anterior mid-cingulate cortex (aMCC) regions implicated in pain perception and affective modulation and had positive correlations between light-induced pain ratings and BOLD activity in these cortical areas. Interestingly, topical anesthetic application significantly reduced activation within S1 and aMCC, suggesting that even in chronic cases, peripheral input contributes to central pain amplification. These findings reinforce that heightened light sensitivity and persistent pain in DED are accompanied by measurable CNS alterations [23].

Functional imaging has also been used to investigate neural predictors of treatment response. In a US study of 12 veterans with chronic NOP (average weekly ocular pain ≥ 1 for ≥3 months) and photophobia (NPSI-Eye question #9 ≥ 1), fMRI was performed before and 4–6 weeks after botulinum toxin A injections. Responders, those reporting decreased light-induced unpleasantness (*n* = 6, mean score 87.5 ± 10.8→32.0 ± 32.7, *p* = 0.006), exhibited pre-treatment activation within the spinal trigeminal nucleus during light exposure, whereas non-responders, those reporting no change or worsening in unpleasantness (*n* = 6, mean 54.2 ± 38.3→64.2 ± 28.0, *p* = 0.136), did not. This suggests that brainstem activity within nociceptive relay centers, particularly the spinal trigeminal nucleus, may identify patients more likely to benefit from neuromodulatory therapy such as botulinum toxin A injections, providing a potential biomarker for response stratification [85]. Additional fMRI studies have examined the neural effects of optical interventions for photophobia. In 25 individuals with chronic ocular pain (average weekly ocular pain ≥ 1 for ≥3 months) and photophobia (NPSI-Eye question #9 ≥ 1 and/or OSDI question #1 ≥ 1), the use of FL-41 tinted lenses (Axon Optics; Bountiful, UT, USA) significantly decreased light-evoked BOLD responses in bilateral S1/S2, bilateral insula, right temporal pole, anterior cingulate cortex (ACC), precuneus, paracingulate cortices, and bilateral cerebellar hemispheric lobule VI, though some activation persisted [86]. These findings indicate that visual filters can modulate central pain circuitry and fMRI may serve as a sensitive biomarker to monitor the neural efficacy of both pharmacologic and non-pharmacologic therapies.

Collectively, fMRI studies underscore that DED-associated pain and photophobia often extend beyond the ocular surface to involve maladaptive central processing within cortical and subcortical pain networks. Incorporating fMRI into ocular surface research provides mechanistic insight into central sensitization, identifies neuropathic phenotypes, and can guide precision management by predicting therapeutic responsiveness to interventions targeting central pathways.

## 5. Artificial Intelligence (AI) and Analytical Advances

AI-assisted analysis is considered an emerging approach in corneal nerve assessment, functioning primarily as an analytic overlay that can augment multiple structural and functional modalities. Like most other fields, AI and machine learning (ML) methods are revolutionizing corneal nerve assessment by overcoming key limitations of manual image analysis, including time consumption, substantial inter-observer variability, and constraints that limit large-scale and real-time clinical application [87,88,89]. Deep machine learning (DLM) model approaches, particularly convolutional encoder–decoder networks, now achieve segmentation accuracy equal to or exceeding expert graders [88,89,90]. U-Net and its derivatives remain the predominant framework, combining multi-scale feature extraction and spatial skip connections to preserve nerve continuity [88,89,91].

Performance across studies demonstrates clinical-grade precision. A CNN-based model (CNS-Net) designed for subbasal corneal nerve segmentation and evaluation with IVCM achieved high accuracy and fast speed, with an AUC of 0.96 (CI = 0.935–0.983), 96% sensitivity, and 75% specificity in a CNF segmentation task. It demonstrated a processing speed of approximately 32 images per second on IVCM data with relative deviation ratio for CNFL of ~16% compared to manual annotation. Further, U-Net-based CNF and Dice coefficients (DC) segmentation models with downstream morphometry reported approximately 86% sensitivity, 90% specificity, and intraclass correlation coefficients (ICCs) of 0.85–0.95 for nerve number, length, branching, and tortuosity metrics vs. expert manual annotation [89]. Furthermore, multi-scale guidance networks have achieved DC of approximately 88–89% on IVCM datasets [91]. The automated pipelines reduce per-image processing time from minutes to fractions of a second, improve reproducibility with high ICCs compared to human annotators, and match or exceed human segmentation accuracy in head-to-head comparisons [89,90,92]. While these models achieve near-human accuracy in nerve segmentation, subsequent efforts have expanded beyond density and length metrics to capture higher-order morphologic and textural features relevant to disease phenotyping.

AI now extracts higher-order morphometric and textural features, including tortuosity, branching complexity, fractal dimension, microneuromas, and reflectivity heterogeneity, that enrich phenotyping of neuropathic states [93,94,95]. Automated tortuosity quantification algorithms are achieving tortuosity grading accuracy of 97% and image-level pathological discrimination accuracy of 77–86% when aggregating features across multiple images [93]. Beyond tortuosity metrics, geometric biomarkers such as branching point density and fractal dimension differentiate DED from controls and enrich severity signaling. Microneuroma detection, a potential feature for identifying NCP phenotypes, has been implemented using two-stage networks that first segment corneal layers then classify subregions, achieving DC values of ~0.75 for neuroma detection and 0.81 for general nerve classification on IVCM images [92]. Texture analysis pipelines extract reflectivity patterns, signal intensity heterogeneity, and spatial frequency features that capture subtle morphological changes not apparent to human observers, with multiparametric evaluation combining whorl-like nerve patterns, tortuosity, and density metrics showing enhanced discrimination of DED severity [95,96]. Correlation studies demonstrated that automated tortuosity, branching density, and texture metrics were associated with patient symptom scores, disease severity classifications, and functional outcomes including esthesiometry measurements, providing richer phenotypic information than traditional nerve density alone [93,94,95].

Going forward, machine learning can leverage multimodal integration that merges structural, functional, and clinical data across modalities to improve diagnostic accuracy and clinical utility [91,96]. For instance, studies linking structural nerve changes to functional outcomes have demonstrated that IVCM-derived metrics such as nerve density, tortuosity, and branching significantly correlate with corneal sensitivity measured using esthesiometry and with symptom severity scores measured with validated questionnaires such as the OSDI or DEQ5 [89,91,94]. More recent machine learning and multivariate models further support these associations, showing that combinations of morphological features can explain a substantial proportion of variance in patient-reported ocular pain and DED symptoms [94,95].

## 6. Clinical and Therapeutic Implications

Integrated interpretation of corneal nerve structure and function is essential to phenotyping DED and NOP. When used together, structural and functional assessment technologies can help further explain symptom–sign concordance or discordance. Clinically, symptom–sign discordance (severe symptoms with minimal ocular surface findings) should prompt consideration of a neuropathic or mixed phenotype (“pain without stain”) [35,71,97]. Structural assessment may identify nerve loss and morphological abnormalities such as tortuosity and microneuromas, which have been reported as promising biomarkers in neuropathic corneal pain [14,98]. Functional testing captures corneal sensory gain or loss and can reflect peripheral (dys)function, central sensitization, or mixed mechanisms [71,99,100]. Importantly, functional abnormalities are bidirectional. For example, hypoesthesia, particularly in the setting of epithelial disease, supports corneal denervation and increased risk for NK, whereas hyperesthesia may reflect peripheral sensitization at the nociceptor level, central amplification, or both [10,100]. Integration of these results, along with clinical context and response to topical anesthetic when available, can clarify whether symptoms are more consistent with tear film-driven nociception, peripheral neuropathic mechanisms, and/or centrally mediated pain processing and may inform escalation beyond conventional tear film-centered approaches.

Phenotyping into DED or NOP using these structural and functional assessment modalities can help guide treatment selection. Emerging therapies for DED and OSD increasingly target corneal nerve health, aiming to restore both structure and function. Cenegermin (recombinant human nerve growth factor) has shown promise in promoting corneal nerve regeneration and improving symptoms in moderate to severe DED, with recent randomized trials demonstrating significant improvements in patient-reported outcomes and nerve parameters, while ocular signs such as tear production may not show significant changes [101,102]. Autologous serum tears, particularly at higher concentrations (e.g., 50%), have been shown to enhance corneal nerve regeneration, tear stability, and visual acuity in severe DED, especially in immune-mediated cases like SjD [103]. Scleral lenses, while not directly regenerative, provide a protective environment that supports nerve recovery and symptom relief by maintaining a stable ocular surface and reducing mechanical stress [104,105]. Other nerve-targeted interventions include vitamin B1 and mecobalamin supplementation, which have demonstrated improvements in corneal nerve length, width, and neuroma count, as well as symptom relief in randomized controlled trials [106]. Amniotic membrane therapies, both cryopreserved and dehydrated, have also been shown to promote corneal nerve regeneration and improve sensitivity and clinical outcomes in DED [107,108]. Novel agents such as synthetic tear proteins (e.g., lacripep) and peptide mimetics (e.g., PEDF, collagen mimetic peptides) are under investigation for their ability to drive functional sensory reinnervation and restore ocular surface homeostasis, even in the presence of chronic inflammation [109,110,111].

In additional to phenotyping, advances in IVCM and esthesiometry now allow for detailed structural and functional profiling of corneal nerves, enabling a precision medicine approach to DED management. This individualized approach is particularly relevant for patients with NOP or post-surgical DED, where nerve abnormalities may not correlate with traditional clinical signs. Incorporating nerve metrics into clinical decision making can optimize treatment selection, monitor response, and potentially improve long-term outcomes [51,112,113,114]. For example, patients with near-normal baseline CNFL are more likely to experience significant improvements in symptoms and corneal staining following standard therapies, while those with low CNFL may require more aggressive or alternative interventions [51,114]. Changes in nerve metrics may facilitate prediction of clinical behavior across DED subtypes. When comparing topical plasma rich in growth factors (PRGF) to standard therapy in patients with aqueous deficient and evaporative DED, one Spanish retrospective study found no change in nerve morphology in PRGF-treated subjects with evaporative DED, but improved nerve morphology in PRGF-treated subjects with aqueous deficient DED. Symptoms (captured by the OSDI and intensity scales of the Symptoms Analysis in Dry Eye) improved following PRGF in both disease subtypes. These findings, which suggest that the subbasal nerve plexus responds differently to anti-inflammatory therapy depending on DED pathophysiology, may inform a more individualized approach to management [115]. Nerve parameters can also be used to monitor treatment response. Consistently, post-treatment IVCM changes have been shown to align with improvement in both ocular symptoms and exam. In an Italian cohort study, SjD patients treated with topical cyclosporine for 6 months had decreased OSE-graded tortuosity (3.8± 0.1 to 2.2 ± 0.2, *p* < 0.001) corresponding to decreased OSDI (52.9 ± 5.9 to 28.0 ± 6.9, *p* < 0.0001) scores and increased TBUT (4.2 ± 0.5 to 9.1 ± 0.3, *p* < 0.001) [116]. Similarly, after topical administration of autologous serum tears for several months in a case–control study of patients with NOP, post-treatment IVCM revealed increased corneal nerve density and decreased nerve beading and microneuromas, while symptom severity by subjective pain report correspondingly decreased [117]. Taken together, these findings draw attention to corneal nerve characterization as a promising tool in therapeutic decision making.

Functional assessments, such as esthesiometry thresholds, further refine patient stratification, guide the selection of nerve-targeted treatments, and monitor treatment response [118,119]. For instance, a US study involving 17 individuals with moderate to severe DED secondary to chronic ocular GVHD observed that mean Cochet–Bonnet thresholds increased from 3.11 ± 2.38 mm to 5.16 ± 1.26 mm after 12 weeks of 20% autologous serum tears (*p* = 0.001) [120]. Furthermore, a French study of 30 SjD individuals reported improved sensitivity after 6 months of topical cyclosporine 0.05% (5.1→5.6 mm, *p* = 0.03) [121]. These results underscore that corneal nerve function can recover with treatment, highlighting the utility of serial esthesiometry for monitoring therapeutic response.

Automated and AI-assisted image analysis tools are being developed to standardize and streamline the quantification of these biomarkers, reducing observer variability and enabling large-scale studies [122]. The integration of these biomarkers into trial design allows for more precise assessment of therapeutic efficacy, particularly for nerve-targeted and regenerative treatments [95,122].

## 7. Limitations and Gaps

Although rapid advances in imaging and functional technologies have deepened our understanding of corneal nerve biology, significant limitations remain that hinder standardization, scalability, and clinical translation across platforms. A major limitation across both structural and functional modalities is the lack of standardized acquisition and analysis protocols. A systematic review of 195 IVCM studies found wide discrepancies in imaging parameters and incomplete methodological reporting, underscoring the need for unified standards and transparent reporting criteria [123]. These inconsistencies extend to image processing methods, segmentation thresholds, region of interest selection, and averaging strategies, all of which can markedly influence derived nerve metrics. Similarly, functional modalities such as esthesiometry and QST lack standardized calibration and stimulus delivery protocols, leading to inconsistent reproducibility and limited inter-study comparability. Device-related variability represents another barrier to comparability. Most IVCM systems visualize only a 400 × 400 µm field per frame, making consistent sampling of the same corneal area over time difficult without mosaicking. Quantitative comparisons of identical regions imaged in vivo, and processed ex vivo, show that IVCM underestimates several SNP parameters. In healthy corneas evaluated using both large-scale IVCM mosaics and β-III-tubulin immunohistochemistry, histology yielded higher CNFL, CNFD, and CNBD than IVCM, confirming a modality-dependent shortfall [124]. Although normative reference datasets for IVCM derived corneal nerve metrics have been published, including a large multinational study that proposed age adjusted reference ranges for subbasal nerve parameters, these datasets are not yet universally adopted across platforms or institutions [125]. Most reported normative values therefore remain derived from single center cohorts with heterogenous acquisition and analysis protocols resulting in substantial variability across studies.

Additionally, OCT-based corneal nerve imaging faces distinct modality specific constraints that currently limit clinical applicability. Although several studies have demonstrated the ability of OCT derived techniques to visualize corneal nerve parameters, these technologies remain largely investigational. From a practical standpoint, acquisition costs are substantial, with additional recurring software and maintenance expenses. Reliable image acquisition and interpretation also require specialized training and operator expertise, which may further hinder routine clinical implementation. Technical limitations also persist, including speckle noise and insufficient axial resolution to resolve fine terminal nerve branches. Most importantly, large cohort in vivo validation studies in human subjects are lacking. Such studies are necessary to establish reproducibility, clinical relevance, and concordance with established reference standards such as IVCM, as well as to support regulatory acceptance. Until these barriers are addressed, OCT-based corneal nerve imaging should be considered a research tool rather than a validated clinical modality [18,42].

Functional testing devices face similar constraints. Techniques such as esthesiometry, QST, and fMRI requires skilled technicians. Cutaneous QST remains limited by psychophysical variability, inter-individual differences, and the absence of standardized stimulus parameters, all of which reduce reproducibility across laboratories [126]. The lack of normative datasets for ocular pain populations further prevents establishment of diagnostic thresholds or predictive cutoffs [127]. Key barriers exist with fMRI application, including the indirect nature of the BOLD signal, which, combined with heterogeneity in experimental paradigms (e.g., light-evoked or anesthetic-modulated designs), limits reproducibility and cross-study comparability, constraining clinical translation [128,129].

Despite promising advances, AI-assisted corneal nerve assessment faces several translational challenges. These include limited dataset sizes with heterogeneous imaging protocols, incomplete external validation across institutions, and the absence of explainable artificial intelligence approaches that provide interpretable outputs for clinical decision making [88,89,90,130,131,132]. The field requires standardized acquisition protocols, common annotation guidelines, multicenter reference datasets, and harmonized performance reporting metrics to enable fair algorithm comparison, facilitate regulatory review, and support clinical validation studies [130,131,132].

## 8. Future Directions

The development of portable and point-of-care devices for corneal imaging and functional testing is a key future direction, aiming to increase accessibility and facilitate widespread screening and monitoring. Recent advances in miniaturized IVCM and handheld esthesiometers are making it feasible to perform high-resolution nerve assessments outside of specialized centers, potentially enabling earlier diagnosis and more frequent monitoring of disease progression and treatment response. Regarding OCTs, future work should focus on quantitative, longitudinal imaging of corneal innervation in OSD, enabling objective evaluation of disease progression and therapeutic response, and ultimately bridging the gap between research-grade imaging and routine clinical diagnostics.

AI and cloud-based platforms are revolutionizing corneal nerve quantification by overcoming the limitations of manual analysis. Cloud-enabled analysis allows for centralized data processing, remote expert consultation, and integration with electronic health records, paving the way for large-scale, multicenter studies and real-time clinical decision support. Future clinical management integrating structural data (e.g., IVCM metrics), functional measures (e.g., esthesiometry), inflammatory biomarkers (e.g., dendritic cell density), and patient-reported outcomes can provide a comprehensive, personalized overview of disease activity and facilitate precision-based therapy.

Beyond OSD, corneal nerve metrics are emerging as noninvasive biomarkers of systemic disease, including diabetes, multiple sclerosis, and peripheral neuropathies [133,134,135]. Incorporating these assessments into multimodal dashboards could extend their clinical utility, enabling early detection of neuropathic complications, longitudinal tracking of systemic disease progression, and evaluation of treatment response. By bridging ophthalmology, neurology, and pain medicine, such integrative approaches hold promise for personalized, mechanism-based care across both ocular and systemic domains [136,137,138].

## 9. Conclusions

Corneal nerve evaluation has evolved from a niche research focus to a central pillar of understanding and managing DED and related OSDs. Advances in imaging, esthesiometry, quantitative sensory testing, and neuroimaging have illuminated the dual structural and functional dimensions of these conditions, reframing them as neuroinflammatory disorders rather than purely epithelial or tear film abnormalities. These technologies have deepened insight into the mechanisms underlying symptom–sign discordance and have facilitated more precise patient phenotyping across the neurotrophic–neuropathic spectrum and the selection of targeted therapeutic strategies promoting personalized medicine. However, widespread clinical translation remains constrained by the need for standardized acquisition protocols, validated multicenter datasets, and stronger correlations among imaging, sensory function, and patient outcomes.

Future progress will depend on harmonizing these modalities into accessible, clinically actionable workflows. As structural and functional metrics converge within multimodal, AI-assisted platforms, clinicians will be able to diagnose, subtype, and monitor disease with unprecedented precision, advancing toward a truly personalized approach to ocular surface care.

## Figures and Tables

**Table 1:** Structural assessment technologies.

Modality	Target	Principle	Pros	Cons
**IVCM**				
LSCM (HRT-RCM, Heidelberg) [38,39]	Corneal SNP (with option to image deeper stromal nerves in focal series)	LSCM; 670 nm diode laser raster-scanned; 63× high-NA objective	Near-histology SNP imaging (1–2 μm lateral; ~4 μm axial) Captures tortuosity, beading, microneuromas, immune cells Depth scan enables 3D mapping and longitudinal monitoring	Contact imaging (risk of discomfort) Small FOV (~400 × 400 μm) Specialized device and operator training
SSCM (Confoscan-4, Nidek) [39,40,41]	Corneal epithelium and stromal nerves (SNP visible, lower contrast)	SSCM; slit apertures; brighter imaging	Fast, non-contact imaging Brighter than TSCM (collects more light) Acceptable cell and nerve visualization	Lower resolution/contrast vs. LSCM (SNP less distinct)
**OCT**				
UHR-OCT [42]	SNP and larger stromal nerve bundles	Spectral-domain UHR-OCT; broadband light; ~1–2 µm axial	Non-contact, depth-resolved corneal imaging Can visualize hyper-reflective nerve bundles	Lateral resolution coarser than IVCM (fine endings not resolved) Primarily research use
FF-OCT [18]	SNP and stromal nerves (wide-field en face view)	FF-OCT; camera-based parallel en face interferometric imaging	Rapid wide-field en face capture (~1.26 × 1.26 mm) Confocal-like nerve maps support density mapping	Investigational; limited clinical availability * Motion sensitivity; depth limits may affect in vivo imaging *
CF-OCT [19]	SNP (curved-field wide en face imaging)	optically matches the focal plane to corneal curvature to generate wide-field en face maps	Layer-confined, curvature-matched en face imaging Resolves ~2–4 μm nerve fibers; improves morphology assessment	Investigational; specialized hardware and processing * Limited clinical availability and validation *
Micro-OCT [43]	SNP; epithelial cells; stromal nerve trunks	UHR-OCT with microscope optics; ~1 µm axial; ~1.5 µm lateral	Near-histology, non-contact 3D detail Visualizes fine branching and nerve–epithelium interactions	Preclinical focus (animal/ex vivo); limited human data Small scan volumes; high data/processing demands
PS-OCT [43,44]	SNP and stromal nerve orientation (birefringence mapping)	PS-OCT; dual-polarization detection for birefringence contrast from nerves	Adds birefringence-based nerve contrast Maps nerve orientation/trajectories; may aid automated detection	Requires specialized PS-OCT hardware and analysis
**Other Imaging Modalities**				
Multiphoton Laser Microscopy [45,46]	SNP and other corneal layers (typically with fluorescent labeling)	Two-photon excited fluorescence microscopy (deep, high-resolution imaging)	Subcellular 3D resolution; can image nerves and immune cells Lower phototoxicity; supports time-lapse studies	Requires fluorescent dyes/markers Complex, expensive research setup; not routine clinical

Abbreviations: CF = curved field; FF = full field; FOV = field of view; HRT-RCM = Heidelberg Retina Tomograph with Rostock Cornea Module; IVCM = in vivo confocal microscopy; LSCM = laser scanning confocal microscopy; NA = numerical aperture; OCT = optical coherence tomography; PS- = polarization-sensitive; SNP = subbasal nerve plexus; SSCM = slit-scanning confocal microscopy; UHR- = ultra-high resolution. * General limitations; not explicitly stated in review. Note: Tandem scanning confocal microscopy is described historically and omitted here due to limited current use; the review describes it as an earlier commercial IVCM system.

**Table 2:** Functional assessment technologies.

Modality	Target	Principle	Pros	Cons
**Esthesiometers**				
Cochet–Bonnet [20,62]	Corneal surface nerve endings (central cornea)	Contact nylon filament (variable length; mechanical threshold)	Simple, portable, low cost Long-standing clinical standard (historical comparability)	Contact may disturb epithelium/infection risk Mechanical only (no thermal/chemical) Coarse gradations; examiner/patient variability
Gas (Belmonte type) [21,63,64]	Corneal nerve endings (mechanical, thermal, chemical modalities)	Non-contact gas jet with controlled flow, temperature, and CO_2_	Separately tests mechanical, cold, heat, and chemical sensationsComprehensive sensory profile; non-contact delivery	Bulky/specialized; mostly research useLonger/complex testing; thresholds not directly comparable to Cochet–Bonnet
Brill [65,66]	Corneal nerve endings (mechanical sensitivity via air pulse)	Portable air-pulse esthesiometer with adjustable intensity	Non-contact and portable Standardized mechanical stimuli; good repeatability Moderate correlation with Cochet–Bonnet	Limited long-term validation Limited availability (emerging technology)
Swiss Liquid Jet [67,68]	Corneal nerve endings (central mechanical sensitivity)	Non-contact saline microdroplet jet (software controlled; ~1 mbar steps)	Examiner-independent thresholds Wide, finely graded stimulus range detects subtle changes	Prototype/specialized hardware Not widely available clinically
Kerasense [69]	Corneal nerve endings (central mechanical sensitivity)	Single-use sterile nylon filament contact esthesiometer (mm scale)	Rapid chair-side screening; sterile, disposable Good test–retest and inter-operator reproducibility	Mechanical only; limited published validation Subjective response; per-test cost
**Other Functional Modalities**				
Cutaneous QST [70,71,72]	Trigeminal and somatosensory pathways (skin testing)	Psychophysical mechanical, thermal, and vibration thresholds; summation/aftersensations	Assesses broader sensory network beyond cornea Helps distinguish peripheral vs. central sensitization Complementary objective sensory metrics	Indirect for ocular surface pain Time-consuming; specialized tools/training Psychophysical (subjective reporting variability)
fMRI [73,74]	CNS pain-processing networks activated by ocular stimulation	BOLD fMRI response to ocular surface stimulation or pain	Whole-CNS, noninvasive assessment of pain processing Differentiates central mechanisms; research biomarker	Costly; not practical for routine care Motion artifacts during ocular stimulation Investigational rather than diagnostic

Abbreviations: BOLD = blood oxygen level dependent; CNS = central nervous system; fMRI = functional magnetic resonance imaging; QST = quantitative sensory testing.

## Data Availability

No new data were created or analyzed in this study. Data sharing is not applicable to this article.

## Abbreviations

The following abbreviations are used in this manuscript:

DED	Dry eye disease
OSD	Ocular surface disorder
IVCM	In vivo confocal microscopy
OCT	Optical coherence tomography
QST	Quantitative sensory testing
fMRI	Functional magnetic resonance imaging
AI	Artificial intelligence
TFOS	Tear Film and Ocular Surface Society
DEWS	Dry Eye Workshop
SjD	Sjögren’s disease
OSS	Ocular surface staining
OSDI	Ocular Surface Disease Index
NK	Neurotrophic keratopathy
NCP	Neuropathic corneal pain
SNP	Subbasal nerve plexus
TRPV1	Transient receptor potential vanilloid 1
TRPM8	Transient receptor potential melastatin 8
CNS	Central nervous system
NOP	Neuropathic/nociplastic ocular pain
TSCM	Tandem scanning confocal microscope
HRT_RCM	Heidelberg Retinal Tomograph-Rostock Corneal Module
SSCM	Slit-scanning confocal microscope
LSCM	Laser scanning confocal microscope
NA	Numerical aperture
CNFL	Corneal nerve fiber length
CNFD	Corneal nerve fiber density
CNBD	Corneal nerve branch density
ACC	Automated cell count; later used for anterior cingulate cortex
EDE	Evaporative dry eye
MGD-NCP	Meibomian gland dysfunction-related neuropathic corneal pain
AIDE-NCP	Autoimmune-associated neuropathic corneal pain
US	United States
MGD	Meibomian gland dysfunction
CFS	Corneal fluorescein staining
NSDE	Non-Sjögren’s dry eye
OSE	Oliveira-Soto and Efron scale
TBUT	Tear break-up time
AS	Anterior segment
UHR	Ultra-high resolution
FF	Full field
CF	Curved field
OCM	Optical coherence microscopy
PS	Polarization sensitive
DEQ-5	Dry Eye Questionnaire-5
GVHD	Graft-versus-host disease
HPT	Heat pain threshold
CPT	Cold pain threshold
LASIK	Laser-assisted in situ keratomileusis
BOLD	Blood oxygen level-dependent
aMCC	Anterior mid-cingulate cortex
FL-41	FL-41 tinted lenses
ML	Machine learning
DLM	Deep machine learning
CNN	Convolutional neural network
AUC	Area under the curve
CI	Confidence interval
CNF	Corneal nerve fibers
DC	Dive coefficient
ICC	Intraclass correlation coefficient
PEDF	Pigment epithelium-derived factor
